# Cost‐effectiveness analysis of leadless cardiac resynchronization therapy

**DOI:** 10.1111/jce.16102

**Published:** 2023-10-09

**Authors:** Nadeev Wijesuriya, Vishal Mehta, Felicity De Vere, Sandra Howell, Jonathan M. Behar, Andrew Shute, Michael Lee, Paolo Bosco, Steven A. Niederer, Christopher A. Rinaldi

**Affiliations:** ^1^ School of Biomedical Engineering and Imaging Sciences King's College London London UK; ^2^ Department of Cardiology Guy's and St Thomas' NHS Foundation Trust London UK; ^3^ EBR Systems Inc Sunnyvale California USA; ^4^ National Heart and Lung Institute Imperial College London UK

**Keywords:** biventricular pacing/defibrillation, pacemaker‐bradyarrhythmias

## Abstract

**Background:**

The Wireless Stimulation Endocardially for CRT (WiSE‐CRT) system is a novel technology used to treat patients with dyssynchronous heart failure (HF) by providing leadless cardiac resynchronization therapy (CRT). Observational studies have demonstrated its safety and efficacy profile, however, the treatment cost‐effectiveness has not previously been examined.

**Methods:**

A cost‐effectiveness evaluation of the WiSE‐CRT System was performed using a cohort‐based economic model adopting a “proportion in state” structure. In addition to the primary analysis, scenario analyses and sensitivity analyses were performed to test for uncertainty in input parameters. Outcomes were quantified in terms of quality‐adjusted life year (QALY) differences.

**Results:**

The primary analysis demonstrated that treatment with the WiSE‐CRT system is likely to be cost‐effective over a lifetime horizon at a QALY reimbursement threshold of £20 000, with a net monetary benefit (NMB) of £3781 per QALY. Cost‐effectiveness declines at time horizons shorter than 10 years. Sensitivity analyses demonstrated that average system battery life had the largest impact on potential cost‐effectiveness.

**Conclusion:**

Within the model limitations, these findings support the use of WiSE‐CRT in indicated patients from an economic standpoint. However, improving battery technology should be prioritized to maximize cost‐effectiveness in times when health services are under significant financial pressures.

## Graphical abstract

- This economic model demonstrates that the treatment of selected patients with leadless cardiac resynchronization therapy is likely to be cost‐effective.
- The factor most likely to impact overall cost‐effectiveness is system battery longevity. Advancements in battery technology will be beneficial in improving the treatment's economic profile.

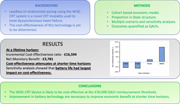

## 1 INTRODUCTION

Cardiac resynchronization therapy (CRT) is an effective treatment for patients with dyssynchronous heart failure (HF).^1^ Conventional CRT involves transvenous lead‐based systems providing biventricular (BiV) pacing from leads in the right ventricle (RV) and in the coronary sinus to achieve epicardial left ventricular (LV) stimulation. Despite its significant success, approximately 30% of patients fail to derive benefit from conventional CRT due to a variety of factors.^2^, ^3^ These patients are known as “CRT non‐responders.” A further 5% of indicated patients are unable to receive conventional CRT due to failure of LV lead implantation, commonly caused by factors such as unsuitable coronary sinus branch anatomy or access site venous occlusion.^4^ Novel CRT technologies are under evaluation for use in this patient cohort, who have limited available treatment options.

The Wireless Stimulation Endocardially for CRT (WiSE‐CRT) system (EBR Systems Inc) is the world's first and only commercially available device which provides BiV CRT by delivering leadless LV endocardial pacing in conjunction with a co‐implanted system which provides RV pacing.^5^ Observational studies^5^, ^6^, ^7^, ^8^, ^9^ and a meta‐analysis^10^ have demonstrated the safety and efficacy of this system. The results from the multicentre prospective SOLVE‐CRT trial will look to add to the existing evidence base with outcomes from a larger patient cohort.^11^

As with many new technologies, WiSE‐CRT commands a higher purchase price than the conventional standard of care (SC) treatment. It is important, therefore, that its' cost‐effectiveness is determined, especially in a time of stalling National Health Service (NHS) budgets, along with rising demand for services and a workforce crisis necessitating increased efficiency.^12^ The cost‐effectiveness of conventional CRT has previously been reported,^13^, ^14^ and importantly, an economic analysis of the ADVANCE‐CRT Registry demonstrated that healthcare costs associated with management of CRT non‐response are amongst the highest for any HF group, thus highlighting the importance of novel CRT technologies.^12^, ^15^ As such, we aimed to conduct an economic analysis of the WiSE‐CRT system.

## 2 METHODS

A cost‐effectiveness evaluation of the WiSE‐CRT system was undertaken with a cohort‐based economic model using Microsoft Excel. The model assessed whether the up‐front costs of the new technology are offset by the expected benefits, with outcomes quantified in terms of quality‐adjusted life years (QALYs) differences.^16^ This economic evaluation was aligned with the reference case of the English National Institute for Health and Care Excellence (NICE).^17^ Costs were applied from the perspective of the NHS in England. Outcomes were quantified in terms of QALYs. Both costs and QALYs were discounted at 3.5% per annum. The reimbursement threshold of £20 000–£30 000 per QALY gained adopted by NICE was used to assess overall cost‐effectiveness. Advanced methodology is presented in the Supplementary Material. Ethical approval was not required.

### 2.1 Model set‐up

The population considered were patients with HF who met criteria for implantation of a CRT device,^18^ plus one of the following criteria: (1) “CRT Non‐Responder”; (2) “Untreated”—de novo CRT was attempted but an LV lead could not be implanted or was not tolerated; (3) “Upgrade”—currently receiving a high burden of RV pacing from a conventional pacemaker (PPM) or implantable cardioverter defibrillator (ICD) device.

**Figure 1:**
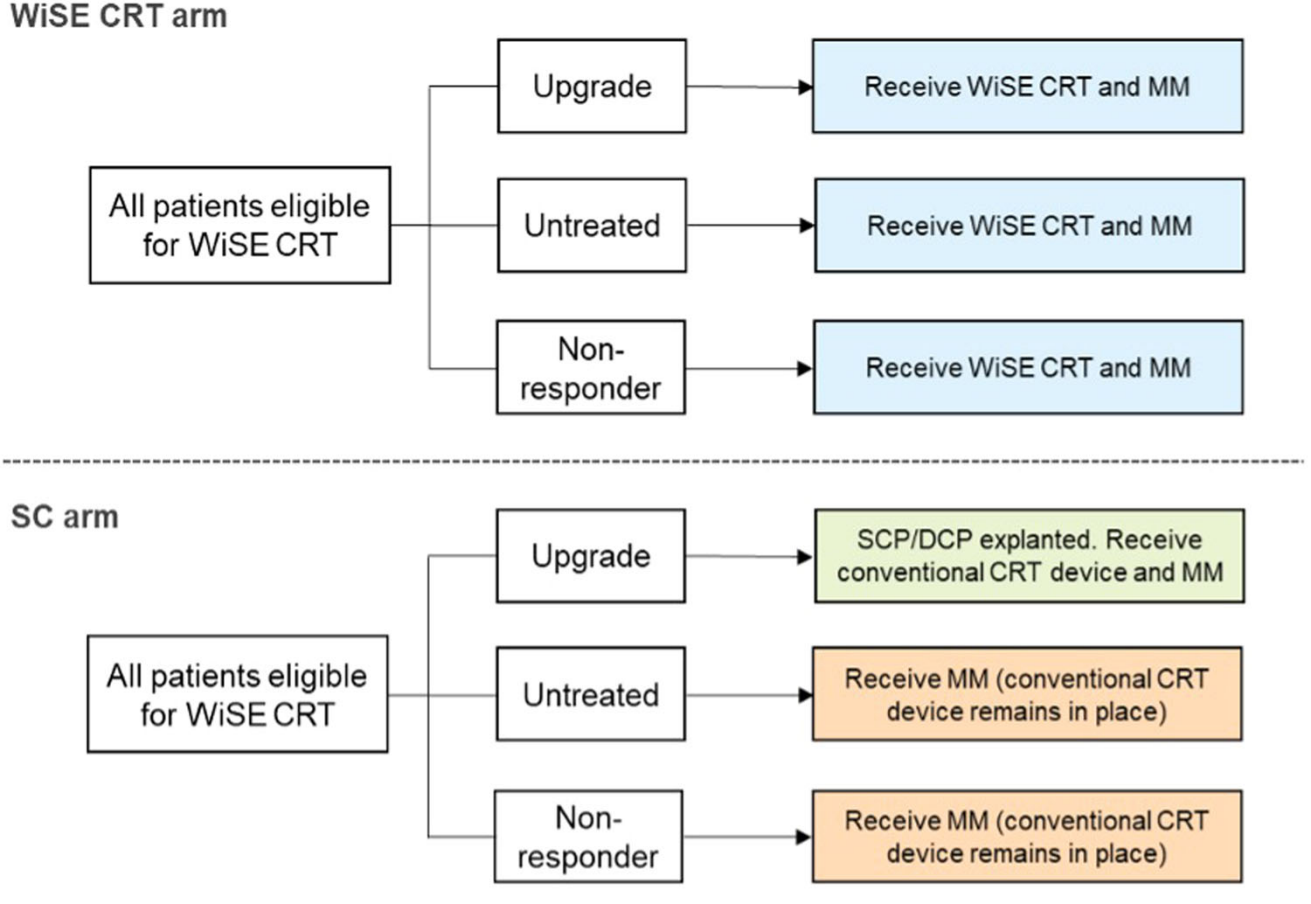
Clinical pathways followed by patients in each arm of the economic model. DCP, dual chamber pacemaker; MM, medical management; SC, standard care; SCP, single chamber pacemaker.

The model adopted a “proportion in state” structure,^19^ where patients could be either alive or dead, with all surviving patients then categorized by New York Heart Association (NYHA) class I–IV. A hypothetical cohort of 1000 patients was followed for a lifetime horizon (Figure 1). The model considers the WiSE‐CRT System as the intervention and SC as the comparator. In both the SC and the WiSE‐CRT arm, all patients have one of four co‐implanted devices in place before the first model cycle: PPM (single chamber pacemaker [SCP] or dual chamber pacemaker [DCP]), ICD, CRT pacemaker (CRTP) or CRT defibrillator (CRTD). In the WiSE‐CRT arm, all patients received the device at the beginning of the first 3‐month cycle. In the SC arm, all patients in the nonresponder and untreated populations did not receive any device in the first cycle. In the upgrade population, patients in the SC arm began with a PPM or ICD, which was upgraded to a CRT device in the first cycle.

The proportion of patients in each NYHA class only changes during the first two model cycles. In subsequent cycles, the proportion of living patients in each NYHA class remained fixed in both arms and persisted for the entire lifetime horizon. This method has been adopted in a previously validated model of pharmacological treatment for HF.^20^ The impact of treatment with WiSE‐CRT on patient outcomes was incorporated via changes in the NYHA class. Costs, resource use and health‐related quality of life (HRQoL) were all assigned based on NYHA class.

All‐cause hospitalizations and device/procedure‐related adverse events (AE) were considered as events influencing costs and resource use. Data to populate the model inputs were sourced from intention‐to‐treat analysis of the SELECT‐LV study^6^ and the WiSE‐CRT postmarket registry^7^ (Supporting Information: Table S1).

Based on a pooled analysis from these studies,^6^, ^7^ patients entered the model at 68 years old, and 80.4% were male. Evidence from published CRT literature was used to generate the proportion of patients in each of the three subpopulations.^2^, ^4^, ^21^ Based on these data, a hypothetical population of interest of patients eligible for WiSE‐CRT was generated, comprising 50% upgrade patients, 7% untreated patients and 43% CRT nonresponders.

### 2.2 Model inputs

The model contains data on the NYHA class mix at baseline and postimplantation derived from the WiSE‐CRT clinical studies (Supporting Information: Table S1). Risk of all‐cause mortality and hospitalization per model cycle was applied based on patients' NYHA class (Supporting Information: Table S2). Rates of device and procedure‐related AEs were derived from the WiSE Clinical data (Supporting Information: Table S1), the 2014 NICE Technology Appraisal of ICDs and CRT (NICE TA314)^22^ and Edwards et al.^23^ WiSE‐CRT device costs were provided by EBR Systems (Supporting Information: Table S3). In this model, the WiSE‐CRT battery is replaced every 4.5 years, and for co‐implanted devices, a 3‐monthly risk of replacement cost was applied per cycle (Supporting Information: Table S4). Costs associated with medical management were applied according to NYHA class (Supporting Information: Table S5). AE costs were modeled as events requiring hospitalization and/or system replacement. The costs and sources from which they were derived are shown in Supporting Information: Table S6 and the average battery life for each device type is shown in Supporting Information: Table S7. Health state utility values were applied according to NYHA class (Supporting Information: Table S8).

### 2.3 Economic analysis

The primary economic outcomes generated by the model were total costs per patient and total QALYs per patient. Based on these outcomes, the model generated an incremental cost‐effectiveness ratio (ICER), representing the cost per QALY gained with WiSE‐CRT, and the net monetary benefit (NMB) at the specified reimbursement threshold (£20 000–£30 000 per QALY gained). NMB is calculated by converting the QALY gain into a monetary value using the reimbursement threshold, which represents the willingness‐to‐pay for the intervention per QALY gained. The incremental cost is then subtracted from this monetary value to generate the NMB. A positive NMB value indicates that WiSE‐CRT is cost‐effective at the threshold value, with larger values indicating greater benefit.

### 2.4 Scenario analysis

To explore the impact on model results of altering certain input parameters of the primary analysis, the following scenarios were analyzed:

1. Exclusion of registry data.^7^ This scenario considers data from the SELECT‐LV study alone.^6^
2. Use of data from the WiSE‐CRT clinical studies^6^, ^7^ to define the proportions of patients in each of the subpopulations, rather than from conventional CRT literature.^2^, ^4^, ^21^
3. Use of 3, 5, and 10‐year time horizons, rather than lifetime.
4. Setting the treatment effect to end in the WiSE‐CRT arm at 10 years post‐implant, at which point at which the NYHA class mix reverts to values in the SC arm.
5. Varying the NYHA class mix at 6 months in the SC arm. In the primary analysis, the assumption around the proportion of patients in NYHA IV is conservative. Therefore, this scenario analyses explored the impact of using the class mix shown in Supporting Information: Table S9.

### 2.5 Sensitivity and sub‐group analysis

To account for first‐order uncertainly, a deterministic sensitivity analysis (DSA) was performed with inputs to the primary analysis varied to their upper and lower 95% confidence intervals to determine which factors had the greatest impact. A probabilistic sensitivity analysis (PSA) was also undertaken with data input not as fixed values but as distributions. The model then runs over 2000 iterations, with each iteration using a different set of values for the inputs and the ICER generated from each was collected, and the spread was examined.

A subgroup analysis was conducted in the upgrade subpopulation, where it was assumed that 100% of the hypothetical cohort of 1000 patients were in this group, and all other aspects of the model remained the same.

## 3 RESULTS

### 3.1 Primary analysis

The results of the primary analysis are summarized in Table 1, with a breakdown of per‐patient costs provided in Table 2. The use of WiSE‐CRT increased total costs due to the cost of the device and associated procedures but was associated with a gain of 1.11 QALYs over a lifetime horizon. At a reimbursement threshold of £20 000 per QALY gained, WiSE‐CRT was estimated to be cost‐effective compared against SC with an ICER of £16 594 and an NMB of £3781.

**Table 1:** Summary of primary analysis.

Result	WiSE‐CRT system	SC	Incremental
Discounted costs per patient	£59 454	£41 029	£18 426
Discounted QALYs per patient	7.24	6.13	1.11
**ICER**	‐	‐	**£16 594**
**NMB (£20 000 per QALY threshold)**	‐	‐	**£3781**
**NMB (£30 000 per QALY threshold)**	‐	‐	**£14 885**

Abbreviations: ICER, incremental cost‐effectiveness ratio, which represents the additional cost to generate one additional QALY with WiSE‐CRT; NBM, net monetary benefit; QALY, quality‐adjusted life year; SC, standard of care.

**Table 2:** Breakdown of per‐patient costs in primary analysis.

Constituent costs (discounted)	WiSE‐CRT System	SC	Incremental
WiSE‐CRT costs (including AEs)	£23 715	£0	£23 715
Other device costs (including AEs)	£19 303	£23 907	−£4604
Medical management costs	£5676	£5609	£68
Hospitalization costs	£10 759	£11 513	−£754

Abbreviations: AEs, adverse events; SC, standard of care.

### 3.2 Scenario analyses

#### 3.2.1 Scenario 1—Exclusion of registry data

In this scenario, the use of the WiSE‐CRT system remained cost‐effective at the £20 000 QALY threshold, with an ICER of £14 000 and NMB of £10 330 (Supporting Information: Table S10).

#### 3.2.2 Scenario 2—Defining subpopulations derived from WiSE‐CRT studies

In this scenario, the subpopulation proportions were changed to reflect the cohorts recruited to WiSE‐CRT clinical studies.^6^, ^7^ This generated a population comprising of 29.3% upgrades, 53.7% untreated, and 17.1% nonresponders. Treatment with WiSE‐CRT remained cost‐effective at the £20 000 threshold, with an ICER of £18 843 and NMB of £1285.

#### 3.2.3 Scenario 3—Use of shorter time horizons

**Figure 2:**
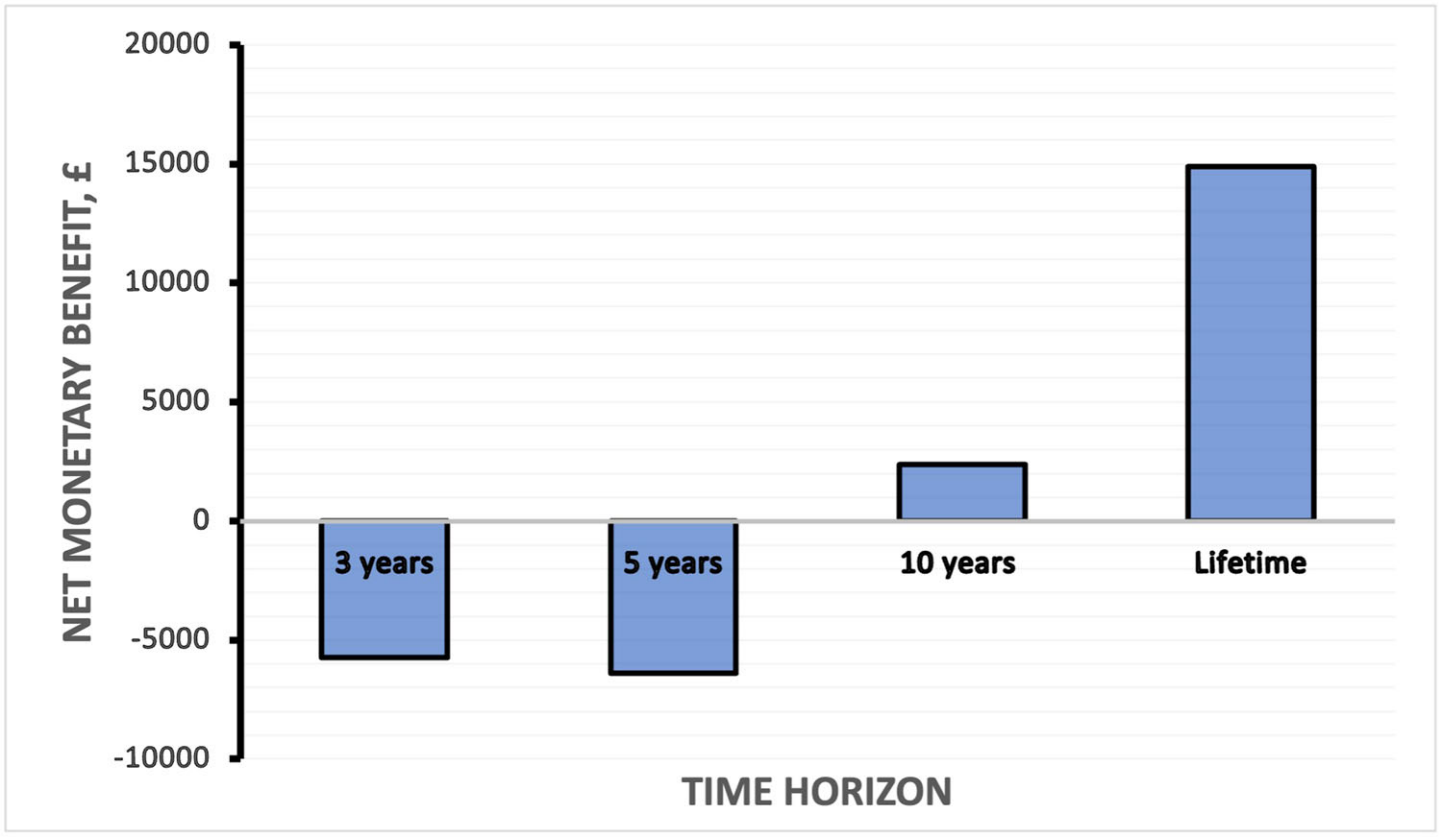
Scenario 3: Graph of time horizon applied to model (x‐axis) versus net monetary benefit (y‐axis, £).

Use of 3, 5, and 10‐year time horizons substantially increased the ICER and decreased the NMB associated with WiSE‐CRT compared with the lifetime horizon. As such, it was not estimated to be cost‐effective when the 3‐year or 5‐year horizons were adopted. The device remained cost effective at the £30 000 threshold at the 10‐year horizon with an NMB of £2384 (Figure 2).

#### 3.2.4 Scenario 4—Treatment effect ending at 10 years

In this scenario, the device remained cost‐effective at the £30 000 threshold, with an NMB of £5329 (Supporting Information: Table S12).

#### 3.2.5 Scenario 5—Varying the NYHA class mix in the SC arm

In this scenario (Supporting Information: as per Table S9), the 6‐month NYHA class mix was adjusted to a less conservative projection of symptom deterioration in the SC arm. The NMB of treatment with WiSE‐CRT is substantially improved to £14 160, with an ICER of £11 690 (Supporting Information: Table S13).

### 3.3 Sensitivity analysis

**Figure 3:**
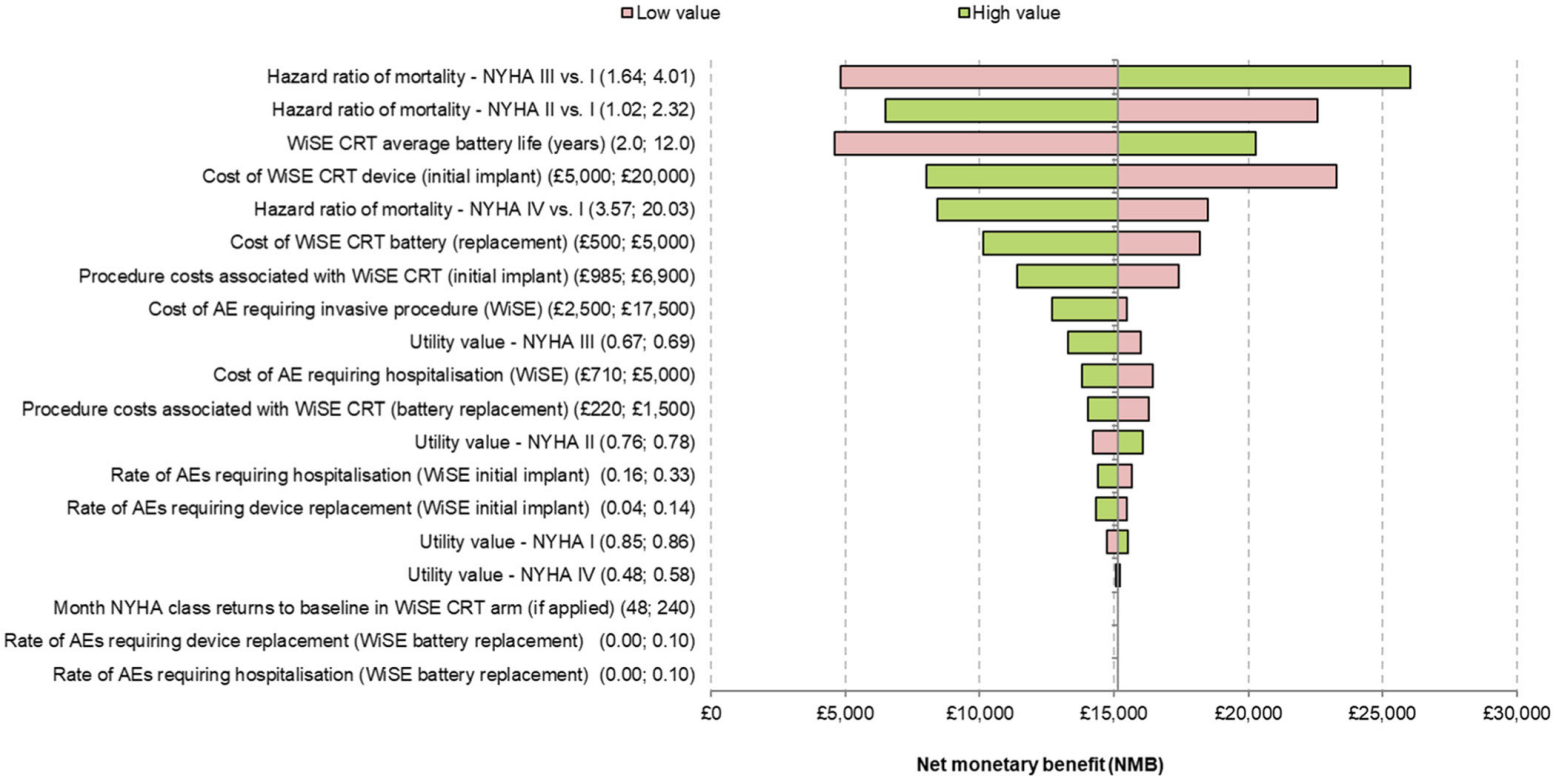
Tornado diagram showing results of deterministic sensitivity analysis using a £30 000 quality‐adjusted life year (QALY) threshold. The numbers in brackets indicate the high and low values for each parameter assessed.

**Figure 4:**
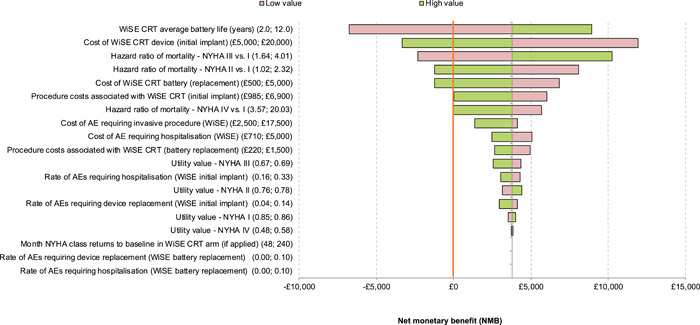
Tornado diagram showing results of deterministic sensitivity analysis using a £20 000 quality‐adjusted life year (QALY) threshold. The numbers in brackets indicate the high and low values for each parameter assessed. The red line represents neutral NMB.

A DSA was performed, with the model output assessed being the NMB at a QALY threshold of £30 000 and £20 000. These are represented in Tornado diagrams. The primary drivers of model results were risk of mortality for people in NYHA classes II and III, the cost of the WiSE‐CRT System (full device) and the average battery life. At the £30 000 threshold (Figure 3), no alteration of any one individual parameter changed the direction of the model's conclusions, that is, the device was cost‐effective if the most unfavorable model inputs were assumed. At the £20 000 threshold (Figure 4), assuming a low average battery life resulted in the WiSE‐CRT not being cost‐effective. At this threshold, unfavorable assumptions around mortality risk in NYHA class II and III and WiSE‐CRT system cost would also drive changes in the direction of the model's conclusions.

**Figure 5:**
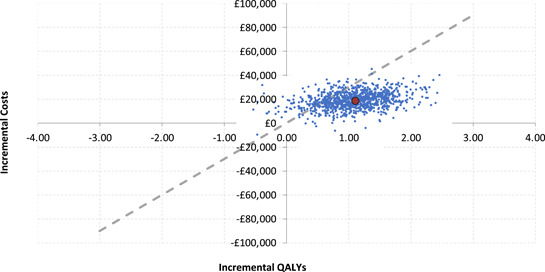
Scatterplot showing the results of probabilistic sensitivity analysis at a £30 000 quality‐adjusted life year (QALY) threshold. Each dot represents an iteration around the primary analysis (large red dot). The dashed line represents the reimbursement threshold.

**Figure 6:**
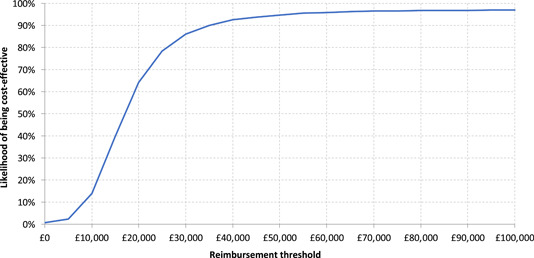
Cost‐effectiveness acceptability curve demonstrating the likelihood of cost‐effectiveness at different reimbursement thresholds.

The PSA results indicate that the estimated likelihood of WiSE‐CRT being cost‐effective is 63.4% at the £20 000 threshold, with a mean ICER of £17 351. This rises to an estimated 85.5% likelihood at a threshold of £30 000. Figure 5 summarizes the average results of the model, and Figure 6 shows the cost‐effectiveness acceptability curve, indicating the estimated likelihood that treatment with WiSE‐CRT is cost‐effective at various reimbursement thresholds.

### 3.4 Subgroup analysis of upgrade patients

In an upgrade population, WiSE‐CRT was projected to be highly cost‐effective with an ICER of £11 863, translating to an NMB of £6242 at the £20 000 threshold (Supporting Information: Table S14). This favorable projection was driven by increased costs of CRT devices compared with standard ICD/PPM devices in the SC arm (Supporting Information: Table S15). PSA estimated likelihoods of cost‐effectiveness of 70.7% and 82.3% at the £20 000 and £30 000 thresholds, respectively.

## 4 DISCUSSION

This economic analysis, including multiple scenario and sensitivity analyses to combat uncertainty, demonstrates that treatment with the WiSE‐CRT device is likely to be cost‐effective at a QALY threshold of £20 000 using a lifetime horizon and at a threshold of £30 000 using a 10‐year horizon. This supports the use of this device for the treatment of patients who are either nonresponders to conventional CRT or who are unable to be treated with conventional CRT. Nevertheless, life‐limiting conditions such as malignancy or noncardiac organ failure should be taken into account when considering treatment, as cost‐effectiveness declines if prognosis is limited to less than 10 years.

The subgroup analysis demonstrates that WiSE‐CRT may be particularly cost‐effective in patients requiring an upgrade from bradycardia pacing to CRT. This supports observational clinical data published by Sidhu et al.,^24^ who reported outcomes of 104 patients receiving an upgrade with WiSE‐CRT compared with 121 consecutive patients who received conventional CRT upgrades. They reported that at 6 months, both groups showed similar improvements in the clinical composite score and reduction in LV end‐systolic volume.^24^ This supports a low threshold being adopted to treating with WiSE‐CRT when a conventional CRT upgrade is unfavorable due to patient factors. Cost‐effectiveness is increasingly probable in patients where alternative options involve high‐risk and costly system revisions or extractions in situations such as venous occlusion or high transvenous lead burden.

A significant result generated from this model was the large impact that battery life has on cost‐effectiveness due to the cumulative costs of multiple generator changes in the WiSE‐CRT arm compared with SC. This is unsurprising, given that economic analyses have demonstrated that extension of traditional transvenous device longevity can lead to savings of approximately 29%–34%.^25^ We believe that this finding should motivate the advancement of battery technologies, perhaps through generators with improved battery life expectancy or rechargeable battery.^26^

It should be noted here that significant improvements to CRTP/CRTD battery longevities with newer generation devices would attenuate the cost‐effectiveness of WiSE‐CRT in the upgrade population. Undertaking further economic analyses will be necessary in time to reflect the probable improved battery performance of both leadless and SC technologies.

In the same vein, whilst biventricular CRT is currently SC treatment for dyssynchronous HF, conduction system pacing (CSP) has the potential to change the landscape of the field.^27^, ^28^, ^29^ Again, future cost‐effectiveness studies will be valuable in evaluating the overall socioeconomic impact of the novel CRT modalities once more long‐term and comparative data is available.

### 4.1 Model limitations

Despite uncertainty being tested using scenario and sensitivity analysis, a number of assumptions and limitations remain in this model. The first simplifying assumption is that the proportions of patients in each NYHA class become fixed after 6 months and remain constant for the duration of the model time horizon, which may not reflect clinical reality. This method for modeling NYHA class has been used and clinically validated in a previous model in HF.^20^ Long‐term follow‐up data is not available from WiSE‐CRT clinical studies. As such, it is not known whether long‐term clinical outcomes with WiSE‐CRT treatment diverge from SC or become more similar. This assumption was tested in Scenario 3, where the treatment effect was stopped at 10 years post‐implant, resulting in reduced cost‐effectiveness. Conversely, in Scenario 5, where the assumption was that outcomes diverged between WiSE‐CRT and SC at 6 months, cost‐effectiveness was substantially improved. To inform future economic analyses, long‐term follow‐up data for WiSE‐CRT patients is crucial, as well as head‐to‐head comparisons in randomized clinical trials. Importantly, long‐term follow‐up data will also improve our estimation of the WiSE‐CRT battery life, which is a major driver of cost‐effectiveness.

A further assumption relates to the relative effects of NYHA class on adverse clinical outcomes. Hazard ratios used to calculate rates of mortality and hospitalization were derived from Ahmed et al.^30^ With improved HF therapies, the hazard ratios for mortality and hospitalization in NYHA II–IV may now be lower. If this is the case, the cost‐effectiveness of WiSE‐CRT may be overestimated by falsely inflated AE costs in higher NYHA classes, which disproportionately affects the SC arm. This is reflected in the DSA, which shows that lower hazard ratios in NYHA II/III patients substantially reduced the WiSE‐CRT NMB, despite remaining cost‐effective at the £30 000 threshold.

In addition, an assumption was made that there was no difference in the treatment efficacy of WiSE‐CRT between the nonresponder, untreated and upgrade populations. This assumption may be accurate, with studies demonstrating that the echocardiographic response rate to WiSE‐CRT in both a nonresponder cohort and a pooled cohort was approximately 50%.^10^, ^31^ This is a small sample size, however, and studies of alternative novel CRT modalities such as conduction system pacing have indicated that the nonresponder population may exhibit a poor clinical response to any form of pacing, possibly due to a more aggressive HF phenotype.^32^ Further evidence on this is awaited from the SOLVE‐CRT trial,^11^ which projects to recruit the largest cohort of nonresponders studied to date.

It should also be noted here that the term “non‐response” has recently been the object of criticism, in view of a lack of a consensus definition between studies and frequent disparities in studies between echocardiographic and clinical response.^33^ In this model, non‐response was defined as deterioration of NYHA class following 6 months of treatment with conventional CRT, which was the definition used in both the SELECT‐LV and Registry studies.

Finally, a simplifying assumption was made that device follow‐up costs were identical in the WiSE‐CRT and SC arms. The financial impact of remote monitoring, which is not yet available for WiSE‐CRT but is being increasingly used for conventional devices, was not incorporated into this model due to the complexity arising from significant regional heterogeneity in access and reimbursement tariffs for this service.^34^ While the availability of remote monitoring may favor the SC treatment, device follow‐up represents a relatively small proportion of total costs and, therefore, would be unlikely to change the direction of conclusions of this model.

## 5 CONCLUSIONS

This economic analysis suggests that the WiSE‐CRT device is likely to be cost‐effective at the £30 000 QALY reimbursement threshold. These results should be interpreted in the context of the model's limitations, including favorable battery life projections and the use of a lifetime horizon. The main economic benefit may be in the upgrade subpopulation, where the cost of a conventional CRT device is offset. Long‐term and comparative data will better inform future analyses.

## Supporting information

Supporting information.

## Data Availability

The data that support the findings of this study are available from the corresponding author upon reasonable request.
