# Supplementary material for: Cost‐effectiveness analysis of leadless cardiac resynchronization therapy
Source: J Cardiovasc Electrophysiol. 2023 Oct 9;34(12):2590–8. doi: 10.1111/jce.16102 (PMC10946454; doi:10.1111/jce.16102)
Supplement: Supplementary file 1 — Supporting information. [file JCE-34-2590-s001.docx]

**Cost-effectiveness analysis of leadless cardiac resynchronization therapy for the treatment of heart failure: Supplementary Material**

**Advanced Methodology**

***Incorporation of treatment efficacy***

The impact of treatment with the WiSE-CRT system on patient outcomes was incorporated via NYHA functional classes. The model contains data on the NYHA class mix at baseline and post-implantation derived from the WiSE-CRT clinical studies (Table S1). In the SC arm, the NYHA class mix is fixed at baseline values from the WiSE-CRT arm, in the absence of published comparative data examining WiSE-CRT versus SC.

**Table S1** – Model inputs for cost-effectiveness analysis. Blue shaded column is the pooled population for the main analysis.

| Parameter | Value | | | |
| --- | --- | --- | --- | --- |
|  | SELECT-LV, all patients | SELECT-LV, upgrades | SELECT-LV + registry, all patients | SELECT-LV + registry, upgrades |
| ***General*** | | | | |
| Number of patients in study | 35 | 3 | 123 | 36 |
| Baseline age | 65 | 69 | 68 | 69 |
| % male | 85.7% | 66.7% | 80.4% | 78.8% |
| ***Subpopulations*** | | | | |
| % ‘non-responders’ | 28.6% | 0.0% | 17.1% | 0.0% |
| % ‘untreated’ | 62.9% | 0.0% | 53.7% | 0.0% |
| % ‘upgrade’ | 8.6% | 100.0% | 29.3% | 100.0% |
| ***Co-implanted devices*** | | | | |
| % SCP/DCP | 2.86% | 33.33% | 12.15% | 28.00% |
| % ICD | 14.29% | 33.33% | 28.04% | 44.00% |
| % CRT-P | 17.14% | 0.00% | 13.08% | 8.00% |
| % CRT-D | 65.71% | 33.33% | 46.73% | 20.00% |
| ***NYHA class mix at baseline*** | | | | |
| % NYHA I | 2.86% | * | 2.44% | 5.56% |
| % NYHA II | 22.86% | * | 33.33% | 50.00% |
| % NYHA III | 74.29% | * | 64.23% | 44.44% |
| % NYHA IV | 0.00% | * | 0.00% | 0.00% |
| ***NYHA class mix at six months post-implantation*** | | | | |
| % NYHA I | 24.24% | * | 16.48% | 15.38% |
| % NYHA II | 66.67% | * | 63.74% | 69.23% |
| % NYHA III | 3.03% | * | 15.38% | 11.54% |
| % NYHA IV | 6.06% | * | 4.40% | 3.85% |
| ***Hospitalisation rates in initial cycle post-implantation*** | | | | |
| NYHA I | *Due to low patient numbers in NYHA I in the WiSE-CRT clinical studies, the general population all-cause hospitalisation rate of 0.07, derived from Ahmed et al was applied in the initial cycle for NYHA I patients in all model scenarios.*^1^ | | | |
| NYHA II | 0.3750 | * | 0.2195 | * |
| NYHA III | 0.5769 | * | 0.3544 | * |
| NYHA IV | *No patients were in NYHA IV at baseline in the clinical data so an assumption was used**** | | | |
| ***Hospitalisation rates in subsequent cycles*** | | | | |
| NYHA I | 0.0000 | * | 0.0000 | * |
| NYHA II | 0.1250 | * | 0.0244 | * |
| NYHA III | 0.3077 | * | 0.1013 | * |
| NYHA IV | *No patients were NYHA IV at baseline in the WiSE-CRT clinical studies and, therefore the hospitalisation rate is assumed to be double that of NYHA, based on Mealing et al.*^2^ | | | |
| ***Device/procedure-related adverse events – initial implant*** | | | | |
| Requiring hospitalisation | 0.45 | * | 0.23 | * |
| Requiring device/battery replacement | 0.20 | * | 0.07 | * |
| ***Costs and resource use*** | | | | |
| WiSE CRT average battery life | 4.5 years | | | |
| Cost of WiSE CRT System | £13,000 | | | |
| Cost of WiSE CRT battery | £2,200 | | | |
| Average surgery time – initial implant | 114 minutes (1.9 hours)  *This value came from the pooled analysis of SELECT-LV and registry data, but was used in all model scenarios.* | | | |
| Average surgery time – battery replacement | 30 minutes (0.5 hours)  *Estimate provided by EBR Systems.* | | | |

***Mortality and Hospitalisation***

A risk of all-cause mortality and hospitalisation per model cycle is applied based on patients’ NYHA class. It was assumed that patients in NYHA I had the same risk of all-cause mortality and hospitalisation as the English general population. Hazard ratios from Ahmed et al^1^ were used to calculate all-cause mortality (Table S2) and hospitalisation (Table S3) in the higher NYHA classes. Annual risks of mortality at each age of the model cohort were taken from the Office of National Statistics life tables.^3^ All-cause hospitalisation rates in the general population were calculated using recent Hospital Episode Statistics data.^4^

Table S2 – Hazard ratios of all-cause mortality and all-cause hospitalisations by

NYHA class derived from Ahmed et al.^1^

| NYHA class | Hazard ratio [95% CI] | |
| --- | --- | --- |
|  | **All-cause mortality** | **All-cause hospitalisations** |
| NYHA II (vs I) | 1.54 [1.02-2.32] | 1.23 [1.00-1.52] |
| NYHA III (vs I) | 2.56 [1.64-4.01] | 1.71 [1.33-2.18] |
| NYHA IV (vs I) | 8.46 [3.57-20.03] | 3.40 [1.69-6.84] |

**Table S3** – Costs associated with WiSE-CRT implant and battery replacement procedures

| Parameter | Initial implant | Battery replacement | Source |
| --- | --- | --- | --- |
| Procedure time (minutes) | 114 | 30 | EBR Systems. Pooled analysis of the WiSE registry and SELECT-LV data for initial implant.^5,6^ Estimate for replacement. |
| Theatre costs (per hour)* | £1,484 | £1,484 | iSD Scotland, 2017.^7^ Direct cost per hour for cardiology specialty. Includes staffing and supplies |
| Proportion of patients receiving inpatient care during procedure | 25% | N/A | Assumption informed by discussions with EBR Systems. |
| Proportion of patients receiving inpatient care after procedure | 50% | 25% | Assumption informed by discussions with EBR Systems. |
| Inpatient care costs (per day) | £510.50 | £510.50 | NHS Reference Costs. Weighted average of elective inpatient excess bed days for all implantations of cardiac devices. |
| Clinician training costs (per procedure) | £0.86 | N/A | Assuming that 8 hours of clinician time are required to attend training (costed at £107 per hour^8^) and they will perform approximately 2 procedures per week over 10 remaining years of working life. |
| **Total Procedure Costs** | **£3,302** | **£870** | Calculated based on the above costs and resource use. |

***Device/Procedure related adverse events***

The model included AEs associated with each of the devices. In the WiSE-CRT arm, these were modelled as rates of events requiring hospitalisation or device replacement, either at time of implant or generator replacement. Rates of each of these type of events were derived from the WiSE-CRT clinical data (Table S1).

For the co-implanted and SC devices, the model included implant failure, peri-operative complications, infections and lead problems, in line with the rates of AEs reported in the 2014 NICE Technology Appraisal of ICDs and CRT (NICE TA314)^9^ and Edwards et al.^10^

***Device cost incorporation and resource use***

Device costs were provided by EBR Systems. These are £13,000 for the full WiSE-CRT System, and £2,200 for the battery only. In this model the WiSE-CRT battery is replaced every 4.5 years. Table S3 shows the constituent costs and resource use for each type of procedure.

The co-implanted devices and associated procedures in the WiSE-CRT arm were costed using information from the published literature.^8,9,11^ Table S4 shows the device and procedure costs applied in the model, along with the sources from which they were derived. The same costs are applied to devices in the SC arm.

In upgrade patients receiving SC, a conventional CRTP or CRTD device is inserted at the beginning of the first model cycle. It is assumed that 25% of upgrade patients receive CRTP and 75% CRTD in SC. The model also includes an additional cost associated with explant of the existing device in these patients, derived from theatre costs reported by iDS Scotland.^7^

**Table S4 –** Co-implanted/SC device procedure costs.

| Device | Unit cost | Source |
| --- | --- | --- |
| ***Device costs*** | | |
| SCP/DCP | £1,712 | Castelnuovo et al.^11^ Mean of reported costs for all types of pacemaker. Inflated to 2017 prices using the HCHS index.^8^ |
| ICD | £10,198 | NICE TA314.^9^ Mean cost of ICD whole system. Inflated to 2017 prices using the HCHS index.^8^ |
| CRT-P | £3,589 | NICE TA314.^9^ Mean cost of CRT-P whole system. Inflated to 2017 prices using the HCHS index.^8^ |
| CRT-D | £12,935 | NICE TA314.^9^ Mean cost of CRT-D whole system. Inflated to 2017 prices using the HCHS index.^8^ |
| ***Procedure costs*** | | |
| SCP/DCP | £4,064 | NHS Reference Costs 2017/18.^12^ Weighted average of elective/non-elective inpatient procedures involving implantation of DCPs [EY05A-EY06E] and SCPs [EY07A-EY08E]. |
| ICD | £4,937 | NHS Reference Costs 2017/18.^12^ Weighted average of elective/non-elective inpatient procedures involving implantation of a cardioverter defibrillator [EY02A-B]. |
| CRT-P | £4,887 | NHS Reference Costs 2017/18.^12^ Weighted average of elective/non-elective inpatient procedures involving implantation of a biventricular pacemaker [EY03Z/EY04A/EY04B]. |
| CRT-D | £4,594 | NHS Reference Costs 2017/18.^12^ Weighted average of elective/non-elective inpatient procedures involving implantation of a cardioverter defibrillator with CRT [EY01A-B]. |

***Medical management cost incorporation***

Costs and resource use associated with medical management were applied according to patients’ NYHA class (Table S5). Two types of cost are included: drugs and outpatient care.

Drugs included in the model, the proportion of patients taking each drug and dosing information were derived from NICE TA314^9^ (table 112 of the assessment report). Costing information was taken from the British National Formulary.^13^

For outpatient care, the model defined a number of disease-related visits per year to a cardiologist, GP or nurse (home visit) for patients in each NYHA class. Unit costs for these visits were taken from PSSRU 2017^8^ for GPs and nurses, and NHS Reference Costs^12^ for cardiologists. The number of disease-related visits per patient per year were sourced from an economic analysis of chronic heart failure reported by Biermann et al.^14^

**Table S5** **–** Drug and outpatient costs per 3-month cycle.

| NYHA class | Drug costs | Outpatient care costs |
| --- | --- | --- |
| NYHA I | £31.29 | £83.58 |
| NYHA II | £42.86 | £106.20 |
| NYHA III | £43.94 | £123.28 |
| NYHA IV | £45.35 | £151.45 |

***Adverse event cost incorporation***

Costs associated with each type of event included in the model were multiplied by the risk of the event in each cycle. The cost of all-cause hospitalisation were set at £2832, in line with NHS Reference Costs (EB03A-E).^12^

With regards to device or procedure-related AEs, costs were applied for events requiring hospitalisation and device/battery replacement in the WiSE-CRT arm. The cost of events requiring hospitalisation was set equal to the cost of all-cause hospitalisation. The model assumes that for system revision AEs, 10% of patients require a full system revision, and 90% require a battery replacement.

For co-implanted/SC devices, costs are applied for implant failure, peri-operative complications, infection, and lead problems. The costs and the sources from which they were derived are shown in Table S6.

**Table 6** **–** Costs of device/procedure-related adverse events associated with co-implanted/SC devices.

| Device | Unit cost of adverse event | | | | Source |
| --- | --- | --- | --- | --- | --- |
|  | **Implant failure** | **Peri-operative complications** | **Infection** | **Lead problems** |  |
| SCP/DCP | £3,944 | £3,944 | £3,944 | £3,944 | Set equal to procedure costs for SCP/DCP (Edwards et al).^10^ |
| ICD | £3,611 | £3,611 | £19,968 | £6,417 | NICE TA314^9^ (Table 111 of the Assessment Report). Inflated to 2017 price year using the HCHS index (PSSRU, 2017)^8^ |
| CRT-P | £5,139 | £5,139 | £13,208 | £5,978 |  |
| CRT-D | £6,980 | £6,980 | £22,707 | £6,415 |  |

***Device replacement cost incorporation***

Costs and resource use associated with generator replacements for each type of device were included (Table S7). All patients in the WiSE-CRT arm undergo a generator replacement at 4.5 years, and every subsequent 4.5-year interval, with the associated cost applied for all living patients. Patients in the WiSE-CRT arm also undergo replacement of their co-implanted device, with a 3-monthly risk of replacement applied per cycle. This is calculated from the average battery life, and multiplied by the device and procedure costs to give a cost associated with replacement in each cycle. In the SC arm, replacements in the non-responder and untreated subpopulations are modelled in the same way as for co-implanted devices in the WiSE-CRT arm. For upgrade patients, who receive conventional CRT in the first model cycle, the full cost of replacement is applied to all living patients in the model cycle when replacement occurs.

**Table S7** **–** Average battery life of each device type.

| Device | Average battery life (years) | Source |
| --- | --- | --- |
| WiSE-CRT | 4.50 | EBR Systems. |
| SCP/DCP | 6.50 | Edwards et al^10^ report that the battery life of a pacemaker is 5-8 years. Mid-point value is used. |
| ICD | 8.20 | NICE TA314^9^ (Table 107 of the Assessment Report). Mean device lifetime. |
| CRT-P | 11.81 |  |
| CRT-D | 7.19 |  |

***Health-related Quality of Life***

Health state utility values were applied according to patients’ NYHA class. All utility values were taken from a study by Gohler et al.^15^ Utility per 3-month cycle in each NYHA class is shown in Table S8.

**Table S8** **–** Utility per 3-month cycle applied, data derived from Gohler et al.^15^

| NYHA class | Utility value | Utility per 3-month cycle |
| --- | --- | --- |
| NYHA I | 0.855 | 0.214 |
| NYHA II | 0.771 | 0.193 |
| NYHA III | 0.673 | 0.168 |
| NYHA IV | 0.532 | 0.133 |

***Economic Analysis***

The following outcomes were generated in each arm of the model and the difference between arms was calculated: Total costs per patient and total QALYs per patient.

Based on these outcomes, the model generated an incremental cost-effectiveness ratio (ICER), representing the cost per QALY gained with WiSE-CRT, and the net monetary benefit (NMB) associated with WiSE-CRT at the specified reimbursement threshold (£20,000 to £30,000 per QALY gained). NMB is calculated by converting the QALY gain into a monetary value using the reimbursement threshold, which represents the willingness-to-pay for the intervention per QALY gained. The incremental cost is then subtracted from this monetary value to generate the NMB. A positive NMB value indicates that WiSE-CRT would benefit the healthcare system at the threshold value, with larger values indicating greater benefit.

***Scenario Analysis***

To explore the impact on model results of altering certain input parameters of the primary analysis, the following scenarios were analysed:

1. Exclusion of registry data. The model considers data from the SELECT-LV study alone,^6^ rather than a pooled analysis.
2. Use of data from the WiSE-CRT clinical studies^5,6^ to define the proportions of patients in each of the subpopulations (non-responder, untreated and upgrade), rather than from conventional CRT literature.^16–18^
3. Use of three, five and 10-year time horizons, rather than lifetime.
4. Setting the treatment effect to end in the WiSE-CRT arm. The point at which the NYHA class mix reverts to values in the SC arm was set at 10 years post-implantation.
5. Varying the NYHA class mix at six months in the SC arm. In the primary analysis, the assumption around the proportion of patients in NYHA IV is quite conservative. Therefore, this scenario analyses explored the impact of using the class mix shown in Table S9.

**Table S9** **–** Proportions of patients in each NYHA class in each SC arm used in scenario analysis.

| NYHA class | % patients in each class at baseline (unchanged in scenario analysis) | % patients in each class at six months (scenario analysis) | Rationale |
| --- | --- | --- | --- |
| NYHA I | 2.4% | 0.0% | All living patients have moved into NYHA II. |
| NYHA II | 33.3% | 25.8% | Patients who were NYHA I at baseline have moved into this class (+2.4%) However, 10% of patients in this class at baseline have moved into NYHA III (-10%) |
| NYHA III | 64.2% | 64.2% | 10% patients who were NYHA II at baseline have moved into this class and 10% of patients in this class at baseline have moved to NYHA IV. Therefore, there is no change. |
| NYHA IV | 0.0% | 10.0% | 10% of patients who were NYHA III at baseline have moved into this class. |

### *Deterministic Sensitivity Analysis (DSA)*

In order to account for first-order uncertainty around the data used for input parameter values, one-way DSA was performed. The parameters that were assessed and results of the DSA are displayed in a tornado diagram. This shows which parameters are the main drivers of model results and the effect that varying these has on the NMB associated with WiSE-CRT. The ranges used to vary the parameters were based on 95% confidence intervals from the literature, with assumptions being made where these were not available.

***Probabilistic Sensitivity Analysis***

In addition to the DSA described above, a probabilistic approach to sensitivity analysis (PSA) was also undertaken. In PSA, the uncertain inputs in the model were each simultaneously selected from a distribution rather than using one fixed value for each input. The model then runs over 2000 iterations, with each iteration using a different set of values for the inputs. The ICER generated from each was collected and the spread was examined.

To generate the input values for each iteration, Dirichlet, gamma, lognormal or beta distributions were fitted to uncertain parameters, depending on the properties of the parameter of interest. The standard errors used to generate probabilistic values were derived from reported 95% confidence intervals wherever possible. Where this information was not available, the standard error was assumed to be equal to 25% of the mean value.

***Subgroup analysis***

A subgroup analysis was conducted in the upgrade subpopulation, where it was assumed that 100% of the hypothetical cohort of 1000 patients were in this group. All other aspects of the model primary analysis remained the same.

**Additional Results Tables**

**Table S10** - Scenario 1: Exclusion of Registry data.^5^ Only data from SELECT-LV Study included in analysis.^6^

| Result | WiSE-CRT System | SC | Incremental |
| --- | --- | --- | --- |
| Discounted costs per patient | £70,246 | £46,143 | £24,103 |
| Discounted QALYs per patient | 8.33 | 6.60 | 1.72 |
| **ICER** | - | - | **£14,000** |
| **NMB (£20,000 per QALY threshold)** | - | - | **£10,330** |
| **NMB (£30,000 per QALY threshold)** | - | - | **£27,547** |

**Table S11 -** Scenario 2: Using WiSE-CRT Clinical trial data to define subpopulation proportions.^5,6^

| Result | WiSE-CRT System | SC | Incremental |
| --- | --- | --- | --- |
| Discounted costs per patient | £59,454 | £38,794 | £20,992 |
| Discounted QALYs per patient | 7.24 | 6.13 | 1.11 |
| **ICER** | - | - | **£18,843** |
| **NMB (£20,000 per QALY threshold)** | - | - | **£1,285** |
| **NMB (£30,000 per QALY threshold)** | - | - | **£12,389** |

**Table S12 -** Scenario 4: Treatment effect of WiSE-CRT ends after 10 years and NYHA class reverts to SC values.

| Result | WiSE CRT System | SC | Incremental |
| --- | --- | --- | --- |
| Discounted costs per patient | £58,622 | £41,029 | £17,593 |
| Discounted QALYs per patient | 6.89 | 6.13 | 0.76 |
| **ICER** | - | - | **£23,025** |
| **NMB (£20,000 per QALY threshold)** | - | - | **-£2,312** |
| **NMB (£30,000 per QALY threshold)** | - | - | **£5,329** |

**Table S13 -** Scenario 5: NYHA class mix in SC arm at 6 months is projected less conservatively (see Table S9 for changes and justification).

| Result | WiSE CRT System | SC | Incremental |
| --- | --- | --- | --- |
| Discounted costs per patient | £59,454 | £39,535 | £19,919 |
| Discounted QALYs per patient | 7.24 | 5.53 | 1.70 |
| **ICER** | - | - | **£11,690** |
| **NMB (£20,000 per QALY threshold)** | - | - | **£14,160** |
| **NMB (£30,000 per QALY threshold)** | - | - | **£31,200** |

**Table S14 -**  Subgroup analysis of upgrade patients.

| Result | WiSE CRT System | SC | Incremental |
| --- | --- | --- | --- |
| Discounted costs per patient | £55,185 | £46,084 | £9,101 |
| Discounted QALYs per patient | 7.09 | 6.33 | 0.77 |
| **ICER** | - | - | **£11,863** |
| **NMB (£20,000 per QALY threshold)** | - | - | **£6,242** |
| **NMB (£30,000 per QALY threshold)** | - | - | **£13,914** |

**Table S15 -** Breakdown of per patient costs in subgroup analysis.

| Constituent costs (discounted) | WiSE-CRT System | SC | Incremental |
| --- | --- | --- | --- |
| WiSE CRT costs (including AEs) | £23,502 | £0 | £23,502 |
| Other device costs (including AEs) | £15,925 | £29,923 | -£13,998 |
| Medical management costs | £5,505 | £5,442 | £62 |
| Hospitalisation costs | £10,253 | £10,719 | -£465 |

**Supplementary Material References**

1. Ahmed A, Aronow WS, Fleg JL. Higher New York Heart Association classes and increased mortality and hospitalization in patients with heart failure and preserved left ventricular function. *Am Heart J*. 2006;151(2):444-450. doi:10.1016/J.AHJ.2005.03.066

2. Mealing S, Woods B, Hawkins N, et al. Cost-effectiveness of implantable cardiac devices in patients with systolic heart failure. *Heart*. 2016;102(21):1742-1749. doi:10.1136/HEARTJNL-2015-308883

3. National life tables, UK - Office for National Statistics. Accessed February 10, 2023. https://www.ons.gov.uk/peoplepopulationandcommunity/birthsdeathsandmarriages/lifeexpectancies/bulletins/nationallifetablesunitedkingdom/2015to2017

4. Hospital Episode Statistics, Admitted Patient Care - England, 2014-15 - NHS Digital. Accessed February 10, 2023. https://digital.nhs.uk/data-and-information/publications/statistical/hospital-admitted-patient-care-activity/hospital-episode-statistics-admitted-patient-care-england-2014-15

5. Sieniewicz BJ, Betts TR, James S, et al. Real-world experience of leadless left ventricular endocardial cardiac resynchronization therapy: A multicenter international registry of the WiSE-CRT pacing system. *Heart Rhythm*. 2020;17(8):1291-1297. doi:10.1016/j.hrthm.2020.03.002

6. Reddy VY, Miller MA, Neuzil P, et al. *Cardiac Resynchronization Therapy With Wireless Left Ventricular Endocardial Pacing The SELECT-LV Study*.; 2017.

7. Finance | Costs | Health Topics | ISD Scotland. Accessed February 15, 2023. https://www.isdscotland.org/Health-Topics/Finance/Costs/

8. Unit Costs of Health and Social Care 2017 | PSSRU. Accessed February 15, 2023. https://www.pssru.ac.uk/project-pages/unit-costs/unit-costs-2017/

9. Overview | Implantable cardioverter defibrillators and cardiac resynchronisation therapy for arrhythmias and heart failure | Guidance | NICE. Accessed February 15, 2023. https://www.nice.org.uk/guidance/ta314

10. Edwards SJ, Karner C, Trevor N, Wakefield V, Salih F. Dual-chamber pacemakers for treating symptomatic bradycardia due to sick sinus syndrome without atrioventricular block: a systematic review and economic evaluation. *Health Technol Assess*. 2015;19(65):1-210. doi:10.3310/HTA19650

11. Castelnuovo E, Stein K, Pitt M, Garside R, Payne E. The effectiveness and cost-effectiveness of dual-chamber pacemakers compared with single-chamber pacemakers for bradycardia due to atrioventricular block or sick sinus syndrome: systematic review and economic evaluation. *Health Technol Assess*. 2005;9(43):1-129. doi:10.3310/HTA9430

12. [ARCHIVED CONTENT] Archived Reference Costs | NHS Improvement. Accessed February 13, 2023. https://webarchive.nationalarchives.gov.uk/ukgwa/20200501111106/https://improvement.nhs.uk/resources/reference-costs/

13. BNF (British National Formulary) | NICE. Accessed February 16, 2023. https://bnf.nice.org.uk/

14. Biermann J, Neumann T, Angermann CE, et al. Economic burden of patients with various etiologies of chronic systolic heart failure analyzed by resource use and costs. *Int J Cardiol*. 2012;156(3):323-325. doi:10.1016/J.IJCARD.2012.01.099

15. Göhler A, Geisler BP, Manne JM, et al. Utility estimates for decision-analytic modeling in chronic heart failure--health states based on New York Heart Association classes and number of rehospitalizations. *Value Health*. 2009;12(1):185-187. doi:10.1111/J.1524-4733.2008.00425.X

16. Brignole M, Auricchio A, Baron-Esquivias G, et al. 2013 ESC Guidelines on cardiac pacing and cardiac resynchronization therapyThe Task Force on cardiac pacing and resynchronization therapy of the European Society of Cardiology (ESC). Developed in collaboration with the European Heart Rhythm Association (EHRA). *Eur Heart J*. 2013;34(29):2281-2329. doi:10.1093/EURHEARTJ/EHT150

17. McAlister FA, Ezekowitz J, Hooton N, et al. Cardiac Resynchronization Therapy for Patients With Left Ventricular Systolic Dysfunction. *JAMA*. 2007;297(22):2502. doi:10.1001/jama.297.22.2502

18. Wouters PC, Vernooy K, Cramer MJ, Prinzen FW, Meine M. Optimizing lead placement for pacing in dyssynchronous heart failure: The patient in the lead. *Heart Rhythm*. 2021;18(6):1024-1032. doi:10.1016/J.HRTHM.2021.02.011
